# Prediagnosis recognition of acute ischemic stroke by artificial intelligence from facial images

**DOI:** 10.1111/acel.14196

**Published:** 2024-06-06

**Authors:** Yiyang Wang, Yunyan Ye, Shengyi Shi, Kehang Mao, Haonan Zheng, Xuguang Chen, Hanting Yan, Yiming Lu, Yong Zhou, Weimin Ye, Jing Ye, Jing‐Dong J. Han

**Affiliations:** ^1^ Peking‐Tsinghua Center for Life Sciences, Academy for Advanced Interdisciplinary Studies, Center for Quantitative Biology (CQB) Peking University Beijing China; ^2^ Key Laboratory of Computational Biology, Shanghai Institute of Nutrition and Health, Chinese Academy of Sciences University of Chinese Academy of Sciences Shanghai China; ^3^ Emergency Department, Ruijin Hospital Shanghai Jiao Tong University School of Medicine Shanghai China; ^4^ Department of Geriatrics, International Laboratory in Hematology and Cancer, Medical Center on Aging of Shanghai Ruijin Hospital, Shanghai Jiao Tong University School of Medicine Ruijin Hospital/CNRS/Inserm/Cote d'Azur University Shanghai China; ^5^ The State Key Laboratory of Medical Genomics Pole Sino‐Francais de Recherche en Sciences Du Vivant et Genomique Shanghai China; ^6^ Clinical Research Institute, Shanghai General Hospital Shanghai Jiao Tong University School of Medicine Shanghai China; ^7^ School of Public Health Fujian Medical University Fuzhou China; ^8^ Department of Medical Epidemiology and Biostatistics Karolinska Institutet Stockholm Sweden; ^9^ Peking University Chengdu Academy for Advanced Interdisciplinary Biotechnologies Chengdu China

**Keywords:** aging, diagnosis model, facial images, stroke

## Abstract

Stroke is a major threat to life and health in modern society, especially in the aging population. Stroke may cause sudden death or severe sequela‐like hemiplegia. Although computed tomography (CT) and magnetic resonance imaging (MRI) are standard diagnosis methods, and artificial intelligence models have been built based on these images, shortage in medical resources and the time and cost of CT/MRI imaging hamper fast detection, thus increasing the severity of stroke. Here, we developed a convolutional neural network model by integrating four networks, Xception, ResNet50, VGG19, and EfficientNetb1, to recognize stroke based on 2D facial images with a cross‐validation area under curve (AUC) of 0.91 within the training set of 185 acute ischemic stroke patients and 551 age‐ and sex‐matched controls, and AUC of 0.82 in an independent data set regardless of age and sex. The model computed stroke probability was quantitatively associated with facial features, various clinical parameters of blood clotting indicators and leukocyte counts, and, more importantly, stroke incidence in the near future. Our real‐time facial image artificial intelligence model can be used to rapidly screen and prediagnose stroke before CT scanning, thus meeting the urgent need in emergency clinics, potentially translatable to routine monitoring.

## Graphical abstract

Prediagnosis model by artificial intelligence and facial images accurately evaluates acute ischemic stroke risk by recognizing abnormal face patterns and associating them with physiological markers and aging.

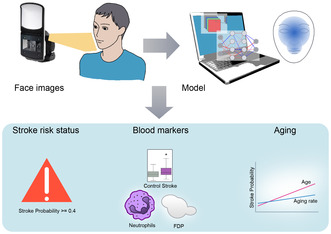

**Abbreviations**
AI: artificial intelligence
AUC: area under curve
CI: confidence interval
CNN: convolutional neural network
CT: computed tomography
FDP: fibrin degradation product
LOESS: locally weighted regression
MRI: magnetic resonance imaging
NET: neutrophil extracellular trap
PCA: principal component analysis
PCC: pearson correlation coefficient
PLS‐DA: partial least square discrimination analysis
RCC: rank correlation coefficient
RGB: red, green, blue
ROC: receiver operation curve
SVM: support vector machine
TIA: transient ischemic attack

## 1 INTRODUCTION

Numerous molecular dysfunctions, such as genome instability, stem‐cell exhaustion, and cellular senescence, manifest during aging (López‐Otín et al., 2013). Consequently, aging is intricately linked to a spectrum of diseases, including macular degeneration (Fleckenstein et al., 2024), cancer (Podolskiy et al., 2016), cardiometabolic multimorbidity (Jiang et al., 2024), stroke thrombectomy outcome (Benali et al., 2024), and atherosclerosis (Tyrrell et al., 2023). The aging process can detrimentally affect the cardiovascular and cerebral vascular systems, thereby exacerbating the progression of ischemic stroke. Research indicates that aging can impair the vasodilation response to acetylcholine in cerebral arteries of WT mice, with even more pronounced effects observed in ACE2 deficiency mice, potentially due to oxidative stress (Peña‐Silva et al., 2012). This impairment may elevate the risk of stroke in aging populations. Furthermore, population studies have demonstrated a significant correlation between shortened telomere length and ischemic stroke, underscoring the pivotal role of aging in stroke etiology (Yetim et al., 2021). Aging can also prompt the emergence of abnormal neutrophils, exacerbating ischemic brain injury (Gullotta et al., 2023; Schulz & Massberg, 2023). Collectively, these evidences solidify stroke as an age‐related disease. Consequently, the timely detection and prevention of stroke not only supports global healthy aging initiatives but also serves to mitigate morbidity and mortality among the elderly population.

Stroke, although considered an acute disease, is well known to have neurovascular problems as the major root cause (Peña‐Silva et al., 2012; Tiedt et al., 2022). For example, chronic inflammation of the blood vessels (Esenwa & Elkind, 2016; Lo et al., 2003) and elevated coagulation alter the general microvessel network, leading to high risk of stroke (Petersen et al., 2018). This central facial area, known as the “central cyanosis zone,” reflects blood flow to the brain. Central cyanosis refers to a bluish discoloration of the lips and mucous membranes inside the mouth that occurs when there is a decrease in the oxygen saturation of the blood under poor blood flow or insufficient oxygen. Changes in the color of these areas can provide valuable information about the oxygenation status of the blood and potentially reflect the overall blood flow to the brain and other vital organs. (McMullen & Patrick, 2013) Moreover, as a consequence of severe stroke, there are numerous neural muscular impairments observable as paralysis on the face and limbs (Schimmel et al., 2017).

The incidence of stroke has continued to climb in recent years, making it the second leading cause of death and creating heavy burdens for both individuals and society as a whole (Feigin et al., 2021). Lifestyle (Pandian et al., 2018) and genetics (Boehme et al., 2017; Georgakis & Gill, 2021; Malik et al., 2015; Markus & Bevan, 2014; Zheng et al., 2019) are both contributing factors to stroke risk. Due to the complexity and severity of the condition, early diagnosis is critical for effective stroke treatment and recovery (Zachrison & Schwamm, 2022). In the recent three decades, the annual number of stroke cases and deaths caused by stroke has particularly increased among individuals over 70 years of age (Feigin et al., 2021). Moreover, the recent COVID‐19 pandemic has further elevated stroke risk. As much as 78% of COVID‐19 patients suffer from cardiac impairment, and 58% of those with long COVID face an even greater risk of various cardiovascular diseases, including heart failure, dysrhythmias, and stroke (Davis et al., 2023). Therefore, practical and easy methods for monitoring stroke risk are essential for people at risk, such as the elderly and those suffering from long‐term COVID‐19.

Acute stroke diagnosis typically relies on noncontrast computed tomography (CT), CT perfusion imaging, and angiography. However, the limited availability of CT can hinder clinicians from quickly identifying and diagnosing stroke. In such cases, body or facial symptoms, such as severe facial paralysis, may be used for early and rough stroke recognition. Unfortunately, subtle facial symptoms can be difficult to recognize in patients in the early stages of a stroke. Typically, clinicians cannot obtain a standardized quantitative numeric stroke probability value through conventional diagnostic methods, as they primarily rely on symptoms or imaging techniques such as CT or MRI scans. This limitation motivated us to develop a predictive model that can rapidly estimate stroke probability to assist clinicians in their diagnostic process. Artificial intelligence (AI) has been used to reliably detect neurological disorders from facial images, as evidenced by previous studies (Gurovich et al., 2019; Hsieh et al., 2022). Many image‐based methods like combining deep learning and CT (Wu et al., 2021) or electroencephalogram (Boyd et al., 2017; Farid & Djamal, 2021; Kaur et al., 2022) have been proposed to predict stroke. However, these methods are limited by expensive or time‐consuming image generation or special devices like CT or MRI machines; thus, they are not suitable for rapid routine screens that are urgently needed for the general population. Thus, it is still an open question whether symptoms‐associated fast and noninvasive AI methods can be established to early predict stroke or to serve as computer‐aided rapid prediagnosis for stroke at the finger‐tip, even on a daily basis. Our previous studies have already found biological age can be calculated from facial images by linear models (Chen et al., 2015) and more accurately by AI models (Xia et al., 2020). This inspired us to use deep learning to recognize stroke from facial images collected from emergency rooms in hospitals located in Shanghai. Our model input only relies on face images, which are fast‐collected, noninvasive, and affordable. Our model reached an area under the curve (AUC) on the receiver‐operating curve (ROC) of 0.91 in cross‐validation and 0.82 in the independent dataset. The stroke probability reassuringly showed the association between our model and clinical factors for stroke risk, like the coagulation markers fibrin degradation product (FDP) and D‐dimer, and furthermore predicted future high stroke incidence within 2 years. Moreover, our facial recognition model identified several previously uncertain or unknown clinical indicators, such as neutrophil counts, leukocyte counts, and glucose level, as potential auxiliary markers for stroke risk. In summary, we developed an easy and economical method to scan acute ischemic stroke in a timely manner and detect correlated molecular features.

## 2 RESULTS

### 2.1 Model performance

**FIGURE 1:**
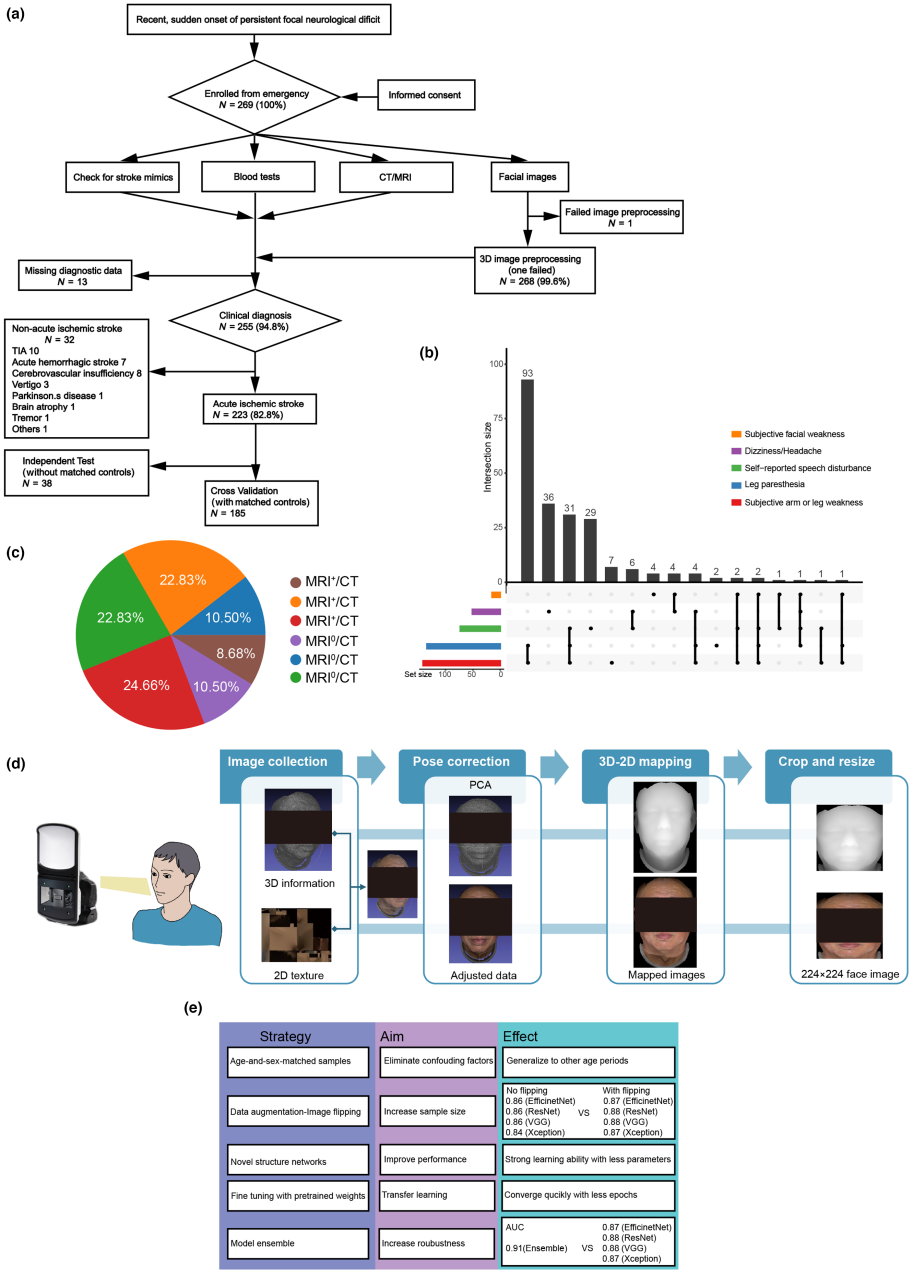
The clinical symptoms and diagnosis process of samples. (a) The flowchart of diagnosis, preprocessing, and utilization of samples. (b) Venn diagram of clinical symptoms of standard pipeline confirmed stroke samples in emergency room diagnosed by clinical experts. (c) Diagnosis status of Ruijin clinical confirmed stroke samples. +: Positive, −: Negative, 0: Missed. (d) The flowchart of image collection and preprocessing. (e) Strategies for model training on relatively small size of samples, with their aims listed and effects tested.

Facial images from patients with highly suspected stroke symptoms, mainly headaches, dizziness, and weakness, were obtained and marked as potential stroke samples by clinical experts (see Section 4, Figure 1a,b). From 2019 to 2021 in Shanghai Ruijing Hospital, 223 acute ischemic stroke samples were diagnosed by MRI/CT‐positive imaging, or when highly suspicious (meet other criteria) but lacking comprehensive medical imaging, diagnosed by symptoms based on a standard diagnosis workflow (2018 China Ischemic Stroke Diagnosis Guidelines) by clinical experts, after dropping all hemorrhage and diseases with similar symptoms, including Parkinson disease or transient ischemic attack (TIA) (Figure 1a,b). Among the 223 imaging‐confirmed ischemic stroke images we used, 219 had electric medical records. In this study, 56.17% and 43.83% of stroke patients were MRI positive and negative, respectively (Figure 1c, Table S1). Of note, all samples have a normal face morphology compared to the naked eye, which is not associated with known neural deficits (see Section 4). A quantitative stroke probability can assist doctors to rapidly evaluate the stroke risk without medical imaging, thus giving treatment priority to critically high‐risk patients. Stoke AI classifier will not only help the patients who do not have CT or MRI but will also help to confirm those who have only CT, or contradictory CT and MRI results, especially given CT has high false negative within 24 h of stroke due to its delayed detection ability. MRI, although more definitive for diagnosis, is more expensive, needs advance appointment, and is unavailable during nonworking hours; hence, it is not preferred.

For model training, we recruited stroke patients from Shanghai Ruijin Hospital and age‐ and sex‐matched negative controls among patients at the same hospital or three other hospitals (Jidong, Majiagou, and Fuqing) (Methods). For model training, we matched age and sex to ensure the model would not be affected by these confounding factors. In total, 185 confirmed stroke patients were kept as the positive group, and 551 non‐stroke or healthy people were matched as the control group (Figure S1a) to build a stroke face recognition program by deep neural networks. The remaining 38 unmatched samples were used as a part of the independent test dataset. Since the larger size of control samples can boost the model robustness and, most importantly, avoid a high false positive rate, we included control samples three times as many as the stroke samples. All samples were preprocessed for generating 224 × 224 images after posture correction (Figure 1d) and randomly split into 10 folds for cross‐validation, followed by data augmentation (see Section 4).

Considering the relatively small sample size for AI approaches, we combine some strategies to boost model performance. These include (1) using only age‐ and sex‐matched normal controls for stroke samples to avoid potential confounding factors, (2) simple data augmentation like image flipping for each sample, (3) utilizing light networks with novel structures like EfficientNet (Tan, 2019) and Xception (Chollet, 2017) to increase generalizability, and (4) taking model ensemble, which gives most boost in model performance according to our monitoring of the AUC at each step (Figure 1e) (see Section 4.4). Furthermore, finetuning in model training using ImageNet pretrained weights helps models to converge (Figure 1e).

**FIGURE 2:**
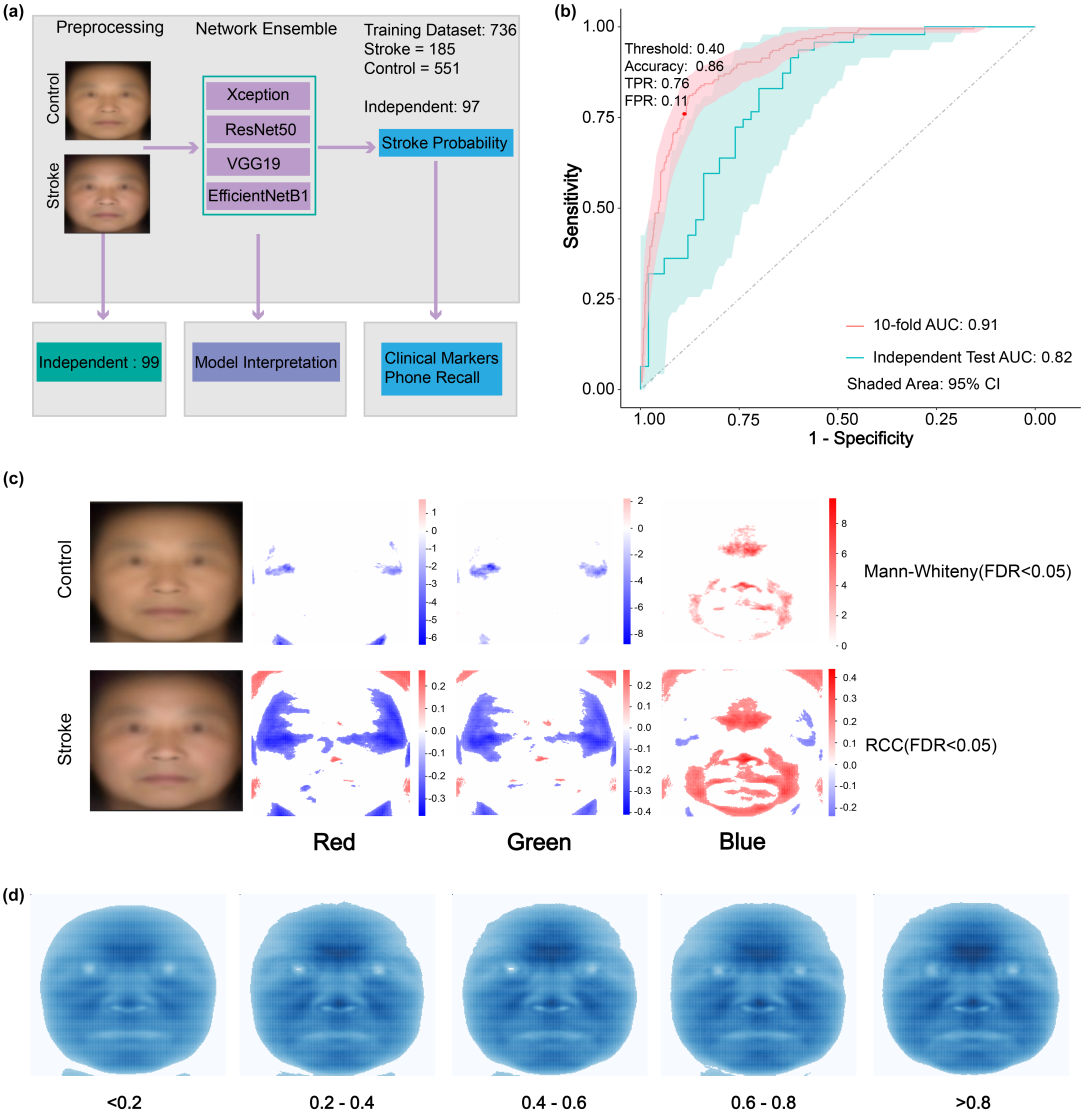
Model performance and interpretation. (a) The flowchart of this study. The texture images, after preprocessing, are fed to CNN networks for predicting stroke probability. The probability is utilized for model interpretation and downstream analysis, including independent cohort validation, model features interpretation, blood indicator association, and predictive power of future stroke incidence. Facial images are synthetic average face. (b) The ROC curves and AUC values on cross‐validation and independent cohorts. The red and green curves correspond to 10‐fold cross‐validation and independent dataset validation, respectively, with shaded areas indicating 95% CI. The red dot indicates the probability threshold of 0.40 used to classify positive and negative groups. (c) Differential pixel maps between model training control and stroke groups in comparison to AI model predicted stroke probability correlated features. The two texture images in the first row are average images of control and stroke groups, respectively; the bottom three maps in the left column are differential maps in which the color intensity corresponds to significance level (FDR <0.05) determined by Mann–Whitney test, with red and blue indicating a higher or lower mean value in stroke group. The bottom three maps in the right column are maps of intensity correlations to AI model calculated stroke probability in red, green, and blue channels, respectively. Color corresponds to significant RCC values (FDR <0.05). All the white pixels indicate non‐significant areas. Facial images are synthetic average face. (d) Average blue channel pixel intensity at different stroke probabilities given by the CNN model. Pixels with values <60 are filtered and visualized as white.

We used four convolutional neural networks (CNN) including EfficientNet (Tan, 2019), Xception (Chollet, 2017), VGG (Simonyan & Zisserman, 2014), and ResNet (He et al., 2016) to train a classifier (see Section 4) (Figure 2a). Cross‐validation was applied to avoid overfitting, and the stroke probability was computed by averaging all augmented replicates of one sample within one model and followed by model ensemble. The Adam optimizer was used for loss optimization while the epoch number was set to 20 (Figure S1c). To unbiasedly evaluate the model performance, we used the AUC metric, which is independent of class size and probability threshold, as well as classification accuracy. Based on the red, green, and blue (RGB) color images without the depth information, the cross‐validation AUC reached 0.91 and an accuracy as 0.86 (95% CI, 0.025) after network ensemble (see Section 4) (Figure 2b). The true positive rate reached 0.76, while the false positive rate was 0.11 under the probability threshold of 0.40. To compare to conventional models, we have applied Partial Least Square Discrimination Analysis (PLS‐DA) and Support Vector Machine (SVM) to the preprocessed texture images. The AUCs of PLS‐DA and SVM are 0.80 and 0.82, respectively (Figure S1d), significantly lower than the AUC of 0.91 attained by the CNN model, demonstrating the superiority of CNN in this task. We further prepared an independent dataset to examine the generalizability of the model. The independent dataset included 38 additional stroke samples and 20 controls from Ruijin Hospital, 9 stroke samples and 6 controls from Jiading hospital, 15 controls from Jidong Hospital, and 9 controls from Majiagou hospital (Figure S1b). Samples in the independent test dataset are not age‐ or sex‐matched, or limited to the same age and sex distribution as the training dataset, so that a good performance on the independent test dataset will indicate the model is generalizable to other independent cohort even when confounded by age, sex, and other unanticipated factors. On this independent dataset, the model reached an AUC of 0.82 and an accuracy of 0.73 (95% CI, ±0.088) (Figure 2b). The high AUC further indicates a robust generalizability of discriminating stroke across different age ranges.

### 2.2 Model interpretation

To interpret the model and identify the facial features that enabled the recognition of the stroke by our CNN model, we compared true control and true stroke groups on all three channels red, green, and blue, and the depth channel of texture images separately by Mann–Whitney U test (see Section 4), and kept the significantly differential pixels (FDR <0.05) for visualization. The red and green channels showed lower mean values in stroke group, while the blue channel showed the most prominent and contiguous features, that is, significantly higher mean values than the control group on mainly ophryon and mouth areas (Figure 2c), consistent with limited blood circulation of ischemic stroke patients, hence darker/bluer center of the face. We further computed the associations between each pixel of R, G, or B channel with the CNN model predicted stroke probability score (Figure 2c). The continuous associations showed a highly similar but more augmented pattern than the differential pattern (Figure 2c,d), suggestive of a more quantitative capability of the AI model than simple differential pattern analysis. The pattern could also be seen by the average blue channel intensity at different stroke probabilities (Figure 2d). Consistent with our CNN model, the PLS‐DA model with two components highlights increased blue channel intensity at the center of the face (Figure S1e), further supporting that central face blue intensity increase is the true biological signal predictive of stroke regardless of the types of models used. The subtle color changes were very hard to be detected by the naked eye but could be detected with high sensitivity by our CNN model. In contrast, the depth information showed no significantly differential pixels (Figure S1f), suggesting 3D information was not very relevant for stoke recognition. The model feature importance calculated by Grad‐Cam (Selvaraju et al., 2020) also showed enrichment at the ophryon and upper nose areas, consistent with the differential pixel maps (Figure S1g). Indeed, adding 3D information to the AI classifier decreased, rather than increased, the classification cross‐validation AUC to 0.88 after the model ensemble. That 2D images allow more accurate CNN model prediction than 3D images may be due to higher noise in the 3D structure than in texture images for stroke detection, thus hindering model performance. This is consistent with the biological origins of the signals. The 2D texture images, in particular the darkening in the blue channel, may reflect blood reduced blood flow to the central face, which reflects that to the brain, the root cause of stroke, while the drooping of the lower face observed on 3D facial contours are more likely a consequence of stroke—neural muscular impairment.

Although the 3D images showed less power for CNN diagnosis model, some distances between 3D facial landmarks were associated with stroke probability. Most of the distances between lower face contour and other facial landmarks showed positive associations with stroke probability (Figure S1h), suggesting that the lower face contour overall droops. Longer distance between nose and mouth are also observed, suggesting a more obvious descent of the mouth (Figure S1h). We also labeled some typical features on face for direct observation (Figure S1i). This area overlapped with the stroke‐associated region in the blue channel in Figure 1d and was consistent with the mouth and eye corners descent on the vertical Y axis shown by stroke probability partial least square regression model on the 3D vertices (Figure S1j).

Among patients with MRI and CT imaging data, 78, 34, and 33 are diagnosed as anterior, posterior, and mixed, respectively. Although the sample size precludes directly classifying the anterior versus posterior, we examined the model‐predicted probability score distribution of each group and found no significant difference between the anterior and posterior groups but a marginally significantly higher stroke probability score for mixed stroke than the anterior stroke, suggestive of a slightly higher severity (Figure S2a). We further computed the CNN model prediction accuracy in the three groups. At the model probability score cutoff at 0.40, the 34 posterior samples show an accuracy of 0.79, while the anterior and mixed stroke show similar accuracies of 0.68 and 0.70. These suggest that although these subtypes may differ in severity, the facial signatures (darker ophryon) are similar, which is indeed the case by comparing the average blue channel signals of the three groups versus samples with low stroke probability (Figure S2b).

### 2.3 Associations of CNN model stroke probability with clinical markers

**FIGURE 3:**
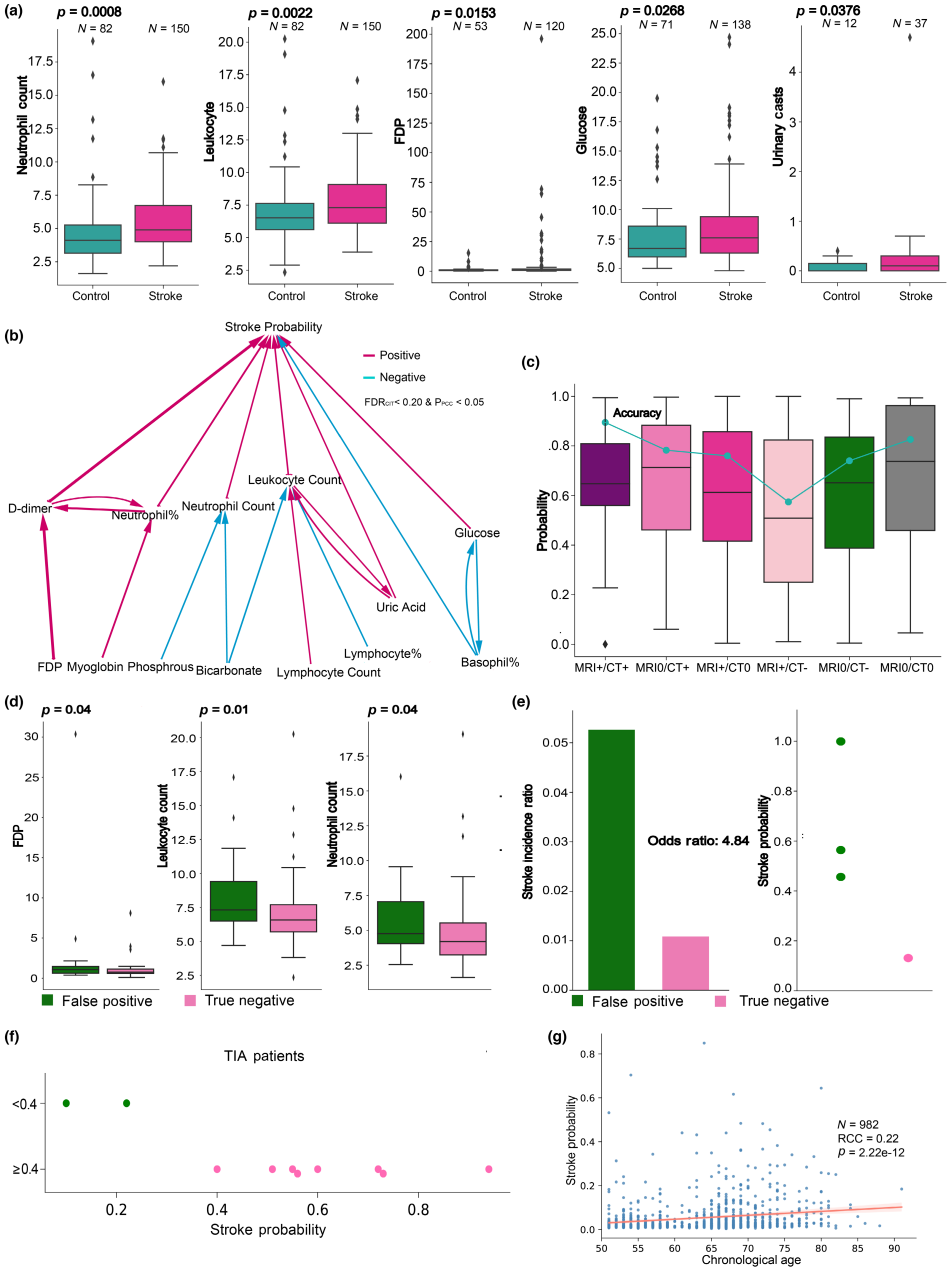
Associations of CNN model stroke probability with clinical metrics. (a) Significantly differential (*p* < 0.05) clinical markers between predicted stroke positive and negative samples separated by CNN model stroke probability 0.4. (b) The causal inference interactions from clinical markers to stroke probability. The line color and width represent the sign of PCC between two nodes and ‐logQ value of the causal test. FDR (*Q* value) of CIT <0.2 together with P value of PCC between each node pair are set to be the threshold. (c) The stroke probability boxplot and accuracy (green dots and line) of each stroke diagnosis status group. +: Positive, −: Negative, 0: Missed. (d) The significant differential markers between the true negative and false positive samples of Ruijin controls. (e) The stroke incidence ratio of false positive and true negative groups of Ruijin control. CNN model stroke probability of the three and one stroke samples in the false positive and true negative groups. (f) Stroke probability distribution of TIA patients. (g) Significant correlation between stroke probability and chronological age for the elderly population (>50 years) of Jidong and Majiagou cohorts.

We next investigated the associations between CNN stroke probability and clinical parameters in the electronic medical records from Ruijin Hospital. Six clinical parameters were significantly differential (*p* < 0.05) in stroke‐negative and ‐positive groups under a stroke probability threshold of 0.4 (Figure 3a, Figure S2c). Among these six biochemistry parameters, some of them have already been demonstrated as an auxiliary biomarker to predict the poor prognosis of stroke, for example, the white blood cell counts and blood glucose level (Chen et al., 2022).

Fibrin degradation product (FDP) as a marker of coagulation showed a significant increase in positive group (Mann–Whitney test *p* = 0.0153). D‐dimer, another coagulation marker, was also detected by computing Spearman rank correlation coefficient (RCC) with stroke probability (Figure S2d). These parameters on circulation functions are consistent with the clinical manifestations of ischemic patients and usually indicate coagulation events (Zeng et al., 2013).

Another group of significant markers was on immune functions, including significantly increased neutrophil count and percentage and leukocyte count in stroke group, with *p* = 0.0008, 0.0460, and 0.0022, respectively (Figure 3a and Figure S2c). Neutrophils can leak from leptomeningeal vessels and reach the brain after ischemic stroke (Perez‐de‐Puig et al., 2015). Other studies found that neutrophils increased in peripheral blood after stroke, which is associated with a worse outcome in patients (Xia et al., 2020). Neutrophophil counts peaked at 12 h after stroke in mice (Xia et al., 2020). Deletion of the PKM2 gene can limit the overactivation of neutrophils, thus improving stroke outcomes, including reduced infarcts and improved cerebral blood flow (Dhanesha et al., 2022). Leukocyte count also increases in hyperacute ischemic stroke patients (Kollikowski et al., 2020), and peripheral polymorphonuclear leukocytes are activated in ischemic stroke (Mo et al., 2013). These are all highly consistent with our results. The last two significantly differential markers were blood metabolites, including increased blood glucose and urinary casts in stroke group, with *p* = 0.0268 and 0.0376, respectively (Figure 3a). Consistent with our findings, hyperglycemia often accompanies patients with ischemic stroke and is related to worse clinical outcomes and larger infarct size (Kruyt et al., 2010). The presence of urinary casts is a marker of kidney function, which is known to affect the pathogenesis of acute ischemic stroke (Chelluboina & Vemuganti, 2019). Spearman RCC also identified two metabolic markers uric acid and creatinine, indicating a worse kidney function to be significantly positively associated with stroke probability (Figure S2d).

The significant markers identified in our differential feature analysis include well‐established blood coagulation markers like FDP, as well as immune cell counts such as neutrophils and leukocytes. These findings are consistent with previous research and align with our clinical knowledge, lending confidence to our results. We further performed the Benjamin–Hochberg correction to the P values for clinical markers. The neutrophil counts, leukocyte counts, and the rank sum of FDP and glucose remain significant after correction (FDR <0.15).

FDP, neutrophil, and leukocyte counts were also correlated with stroke probability, shown by the significant Spearman RCC (Figure S2d), indicating robustness of these associations. Significant negative correlation between mean hemoglobin concentration and stroke probability was also detected by RCC, potentially indicating a low oxygen level in circulation, consistent with the darkened ophryon pattern (Figure S2d). Similarly, the significant markers identified by RCC encompass several typical blood coagulation markers such as FDP and D‐dimer, immune cells including neutrophils and leukocytes, and mean hemoglobin levels that align with previous clinical knowledge, further reinforcing our confidence in the correlation results. After Benjamin–Hochberg correction to the RCC P values, adenosine deaminase, neutrophil counts, FDP, and the rank sum of leukocyte counts and glucose are still significant (FDR <0.2). Thus, for the purpose of identifying potential novel markers, not applying corrections to *p*‐values allows us to cast a wide net and detect promising leads for further investigation, given the inherent trade‐offs between statistical rigor and exploratory analysis in hypothesis generation. Overall, these results demonstrated the ability of our model to detect molecular hints and further confirmed the reliability of our model.

While FDP is a relatively well‐known marker for clinicians, the significant association of neutrophils with stroke may not have garnered as much attention in clinical practice. Therefore, it can indeed be considered a novel indicator with potential utility in clinical settings. Moreover, the identification of two metabolism markers that haven not received significant attention from scientists and clinicians underscores the value of our research. Bringing attention to these markers broadens the scope of clinical evaluation for patients undergoing blood tests and may enhance clinicians' ability to comprehensively assess patients to improve diagnostic accuracy and gain deeper insights into patient health to design tailored effective treatments.

To further infer the interactions among stroke probability‐associated blood factors, we applied causal inference test (Millstein et al., 2009) to clinical markers to identify tripartite regulation networks promoting stroke risk (see Section 4). We constructed two causal networks by setting the significant variables (Figure 3a, Figure S2c) as the mediators, or drivers, and the remaining clinical variables as drivers or mediators, and then visualized them as one combined network. We found that neutrophil percentage formed a positive feedback circuit with D‐dimer and then progressively promoted the stroke probability (Figure 3b). However, increased phosphorous and bicarbonate potentially depleted neutrophil count and alleviated stroke risk. It not only confirmed the importance of neutrophils in ischemic stroke, consistent with the fact that neutrophils were known to affect ischemic stroke by neutrophil extracellular trap (NET) (Denorme et al., 2022) but also reflected a complex regulation network (Figure 3b). Leukocyte count and uric acid also manifested a pattern of positive feedback, consistent with previous results that hyperuricemia often affected neutrophil function (Lowell, 2022) (Figure 3b). Another regulation feedback was between basophil percentage and glucose level. Unlike positive feedback, glucose and basophil percentage repressed another one. Elevated glucose levels can directly increase stroke risk while simultaneously repressing the percentage of basophil, then elevating the stroke risk (Figure 3b). It implied that higher glucose may interact with immune system to increase stroke risk, which is consistent with the fact that hyperglycemia often induces a worse outcome (Kruyt et al., 2010), and highlights the importance of glucose management for ischemic stroke patients. Altogether, the metabolites (Uric acid, Glucose, Bicarbonate) and immune cells (Neutrophil, Basophil, Leukocyte) and their interactions are inferred to regulate the stroke etiology and development. This network reflected the complexity of a non‐linear dynamic biological system. Overall, the above results not only reinforced the reliability of our model but also helped scientists to find novel markers and investigate potential mechanisms behind acute ischemic stroke.

### 2.4 Comparison of AI model to MRI and CT

We further compared our model with the golden standard MRI and CT imaging. After model is trained and the stroke probability assigned to each sample, we further categorized ischemic stroke patients based on their medical imaging status derived from CT/MRI results. Here, our diagnosis model is exclusively based on facial images, with the output being the probability of ischemic stroke using only facial images as input. Model training does not require CT/MRI images. In our study, we utilize CT/MRI images solely to classify different statuses of stroke patients and compare them with the accuracy of our CNN prediction model in downstream analysis. This step is intended to demonstrate the robust performance of our diagnosis model. The stroke samples were divided into three diagnosis statuses: positive (+), negative (−), and missed (0) under MRI or CT. In group with MRI or CT imaging positive, the accuracy of four subgroups indeed decreased from double positive (MRI^+^/CT^+^), single positive (MRI^0^/CT^+^, MRI^+^/CT^0^) to contradictory group (MRI^+^/CT^−^), as expected (Figure 3c). The group MRI^0^/CT^−^ possibly corresponded to a common scenario of false negative imaging of CT for early‐stage stroke, potentially reflecting the pre‐CT diagnosis ability of our model (Figure 3c). Although the last group (MRI^0^/CT^0^) lacked of imaging evidence, the accuracy was surprisingly high, and this was due to very severe symptoms that no MRI or CT was required for diagnosis by clinicians (Figure 3c). Interestingly, the accuracy of MRI^+^/CT^+^ stroke group exceeded 0.80, implying a comparable diagnosis capability of our CNN model to MRI imaging (Figure 3c). Overall, our stroke face recognition CNN model is accurate, robust, convenient, and economical. Using our facial image‐based prediction model, the process of image capture, preprocessing, and prediction together takes only several minutes, in comparison to hours or days of waiting for CT/MRI imaging and analysis.

### 2.5 Forward prediction of stroke risk in the near future

When we further analyzed the false positive and true negative of Ruijin control under a cutoff of 0.40 (Figure S2e), surprisingly, we found that the coagulation makers FDP (*p* = 0.004), leukocyte count (*p* = 0.01) and neutrophil count (*p* = 0.044) also significantly increased in false positive group (Figure 3d). This suggested that the false positives might not be all attributable to model error, but more likely to indicate higher stroke risk; that is, the AI stroke score might be predictive of stroke events in the near future. In order to test this, we conducted patient recalls for the false positives and true negatives in the Ruijin control cohort (visiting patients for diseases other than stroke); 57 and 92 cases were retrieved, respectively. During the period of approximately 2 years from data collection to present, 3 in the 57 false positives have had stroke, while only one in the 92 true negatives has had stroke, with the odds ratio of 4.84 between the two groups (Figure 3e). These remarkable forward risk prediction results by the short‐term longitudinal (rather than cross‐sectional) study suggested the facial image‐based stroke AI predictor was a powerful and convenient tool for stroke screen and risk assessment, and enabled the detection of high‐risk yet non‐stroke individuals for timely and early intervention to prevent disease development.

### 2.6 Potential basis for facial image‐based stroke prediagnosis and early prediction

The significant correlations of our model‐predicted stroke score to the patients' biochemical tests indicative of general vascular problems raised the hypothesis that the link between the facial images and stroke is related to vascular problems. Indeed, we found chronic inflammation and elevated coagulation markers (elevated levels of glucose, FDP, leukocytes, and neutrophils) that alter the general microvessel network manifest a similar phenotype—to the model‐predicted increased stroke probability—blue hue in the center of the face (Figure S3a,b). In addition to the most important facial feature associated with our model's prediction score and accuracy, the bluing of the center of the face (Figure S3c,d), we also observed that high inflammation and coagulation also associated with longer distances between the lower face contour and other landmarks (Figure S3c), drooping eyes and mouth corners (Figure S3d), similar to the contour and shape changes correlated to our model predicted stroke score (Figure S1g,h).

In addition, a TIA, often called a ministroke, is a short period of symptoms similar to those of a stroke caused by a brief blockage of blood flow to the brain, and is predictive of an eventual stroke within a year (https://www.mayoclinic.org/diseases‐conditions/transient‐ischemic‐attack/symptoms‐causes/syc‐20355679). Therefore TIA, sharing a similar cause for stroke—blockage or restriction of blood flow to the brain, but more transient than stroke, can be used to further test our hypothesis. If the facial symptoms reflecting blockage or restriction of blood flow to the brain were the medical basis for early prediction of stroke by our model, we would expect the model gives high stroke score for TIA patients. Indeed, among the 10 TIA patients, 8 are predicted as stroke with stroke scores> = 0.4 and only 2 below 0.4 (Figure 3f). This further suggests that our model, by detecting the root cause, the blood flow restriction to the brain, rather than the direct blockage site, is more powerful than CT or MRI in early prediction of stroke. CT and MRI directly detect the blocked area, while the facial image does not directly image the block sites. It may, however, detect an increase in the blue hue resulting from restriction of the blood flow to the center of the face. Therefore, facial images may not only detect an acute stroke phenotype‐restricted blood flow but can also detect the root cause, a chronic condition reflecting the direct risk factor of stroke, just like high coagulation factors in the blood and ministrokes do; thus the chronic risk factor quantification can have the power of predicting the frequency or incidences of stroke, an acute symptom, in advance for early detection and warning.

## 3 DISCUSSION

Overall, our study successfully identifies a novel facial signature and easily‐accessible biomarker of stroke and constructs a fast diagnosis AI model that may assist clinical decisions and even daily risk evaluation for acute ischemic stroke. The model computed probability is also associated with known blood stroke prognosis biomarkers. Other blood markers, identified by the causal inference test to contribute to our model computed probability, indicate that the facial image builds a connection among several blood markers, which may have application prospects in related disease predictions.

Deep learning models often rely on large sample size. Here, to digitally double the sample size for deep learning, we flipped each image as data augmentation. Furthermore, using light networks with few parameters like Xception and EfficientNet, using pretrained networks by ImageNet data for transfer learning, using model ensembles, using strictly age‐ and sex‐matched controls to exclude age and sex influences, and minimizing false positive rate by training with the controls samples three times as the stroke samples, altogether enabled our deep learning model trained to learn a stroke‐specific signal with low false positive rate on a relative small set (for deep learning models) of stroke samples (Figure 1d). This is confirmed by the high AUC of cross‐validation and independent test dataset validation, as well as the correlation of model prediction score to clinical stroke markers. Our model recognizes the darkening in the blue channel in the center of the face as facial signature of acute stroke, which coincides well with the stroke etiology—block of circulation, and predicts previously known and unknown molecular underpinnings, including coagulation of the blood and neutrophil activation. Furthermore, our stroke probability showed consistency with MRI/CT results, with a comparable capacity to MRI.

In clinical practice, it is important to note that only severe stroke patients typically exhibit obvious facial symptoms such as asymmetry in the eyes and mouth. Most patients cannot be diagnosed solely based on insignificant facial features, leading clinicians to follow a standard diagnosis workflow as outlined in the 2018 China Ischemic Stroke Diagnosis Guidelines. Clinical experts typically assess patients for body symptoms like leg paresthesia and neurological symptoms such as dizziness or headache. Subsequently, CT/MRI imaging, considered the golden standard approach, is employed. This comprehensive diagnostic workflow, while effective, often proves time‐consuming and costly. It also places considerable pressure on doctors tasked with diagnosing and evaluating stroke risk status. Our facial image‐based stroke prediction model not only provides a rapid prediagnosis tool for alleviating the burden on clinicians and for routine stroke screening and monitoring, it also reminds the clinicians that in addition to the well‐known stroke symptoms, the darkening or bluing of the center of the face is another symptom to look for, although it is very hard for the naked eye to detect.

In addition to the well‐established coagulation marker FDP, our analyses have also identified immune markers such as neutrophil counts and metabolism markers like glucose (Figure 3a). Patients with elevated levels of these markers correspond to higher stroke probability. Furthermore, our analysis reveals a higher occurrence ratio of stroke in samples with higher probability (Figure 3e). Given these findings, it is crucial for doctors to pay close attention to patients exhibiting abnormal blood markers and promptly arrange CT/MRI scans and appropriate therapies for suspected cases. Together with our CNN model, such a proactive approach can potentially lead to early detection and intervention, improving patient outcomes and reducing the impact of stroke.

The quantitative nature of these associations also indicated that the model predicted stroke probability was quantitative enough to indicate not only the presence of stroke but also stroke severity and, most importantly, stroke incidence in the near future, which was extremely helpful for the prevention of the deadly disease. Thus, the model could be applied to daily monitoring and dynamically evaluating stroke risk.

Although our training stroke samples were from only one center, the model could be generalized to an independent cohort that included 15 samples from another center, and the saturation analysis indicated that the model performance had approached stability on both training and independent datasets, still with room to increase (Figure S4a). In the future, multicenter collection from different cities and even larger sample size may further boost the model's accuracy and generalizability. Furthermore, with more longitudinal data, a model can be trained to directly learn the probability and time to stroke incidence in the next 5 years. It would be interesting to see whether the model trained on longitudinal data and models trained on cross‐section data converge on the same features associated with stroke probability. Given the future stroke predictive power of our current model, at least some of the features must be shared among models trained on cross‐section and longitudinal data.

Furthermore, as an age‐related condition, the etiology of ischemic stroke encompasses age‐induced vascular dysfunction (Peña‐Silva et al., 2012), immune disorder (Gullotta et al., 2023), and other factors. Our diagnostic model's computed stroke probability shows a significant positive correlation with chronological age among the elderly population in the Jidong and Majiagou cohorts (Figure 3g). Moreover, the aging rates derived from CNN aging clocks trained by perceived age and chronological age (Methods) also manifest significant associations with stroke probability in elderly population of the Jidong cohort (Figure S4b,c). This not only aligns with previous research but also reinforces the reliability of our model. These findings motivate us to explore stroke prevention strategies targeting aging mechanisms, such as the use of anti‐aging medications and regular assessment of aging progression (Wang et al., 2023; Xia et al., 2021). By incorporating our model's potential for assessing stroke risk, we anticipate a reduction in stroke‐related mortality and morbidity, alleviating the societal burden of age‐related diseases and promoting initiatives for healthy aging.

## 4 METHODS

### 4.1 Data collection and preparation

From September 2020 to March 2022, after informed consent, we collected facial images of patients who came to the emergency department of Ruijin Hospital with acute stroke similar symptoms. After completing physical examination, head MRI or CT examination and standard diagnosis and image preprocessing, 223 acute ischemic stroke samples were confirmed by 2018 China Ischemic Stroke Diagnosis Guidelines. We have excluded all the other confounding diseases like hemorrhage, Parkinson's disease, and TIA. To eliminate the confounding effects of age and sex, we finally kept 185 MRI/CT confirmed stroke patients and 551 non‐stroke or healthy people as training dataset. The remaining 38 unmatched stroke patients are taken as a part of the independent test dataset. The inclusion and exclusion criteria of potential patients are listed as follows:

Inclusion criteria: (1) face image information of patients with chief neurological and psychiatric symptoms (dizziness, headache, numbness of limbs, inflexible speech, weakness, physical activity disorder, etc.); (2) clinical symptoms and auxiliary examination results consistent with suspected stroke. (There was no restriction on age, gender, and ethnicity.)

Exclusion criteria: (1) patients who could not cooperate with data collection; (2) patients with unstable vital signs; (3) those unwilling to sign the informed consent form; (4) history of previous facial abnormalities (plastic surgery, tumor, trauma, facial neuromuscular disease, etc.).

The inclusion and exclusion criteria of controls are listed as follows: Individuals visiting the emergency room and diagnosed with non‐stroke‐related diseases are considered controls, along with healthy samples obtained from other centers. Then, only age and sex‐matched samples to stroke patients are used as controls in the training cohort to rule out age when training our models. For independent test set, in order to maximally test the model's generalizability, controls are not age‐ and sex‐matched.

### 4.2 Preprocessing

After the image collection process, each sample corresponds to both a 2D texture image and a 3D face point cloud file. Despite our collection criteria requiring samples to face the exact front, there may still be some pose and position bias present. Therefore, we apply a step called “pose correction” to all samples. This ensures that each face has the same posture and position, thereby eliminating potential confounding factors during model training. Once the pose correction is completed, we can simultaneously map the texture image and the 3D face information with the same resolution. Subsequently, we crop the images to retain only the facial area, followed by resizing them to a standardized size of 224 × 224 pixels. The step‐by‐step procedures for this process are explained as follows with rigorous mathematical formulae described in our previous study (Xia et al., 2020).

1. Initially, we identify the nose tip point by fitting a sphere with a radius of r and a center at (O_X_, O_Y_, O_Z_). For each vertex V, we utilize all vertices within a distance of <1.5 cm to fit a sphere and calculate the loss function. The point with the lowest value of the loss function is defined as the nose tip. The output provides the coordinates of the nose tip.
2. Next, we consider all points around the nose tip within a distance of less than 5 cm to correct the pose, with the objective of aligning every sample to face straight ahead. We apply Principal Component Analysis to these points to obtain the eigenvalues and eigenvectors. The eigenvectors corresponding to the top three eigenvalues are selected as the base vectors of a new coordinate system. Subsequently, the three‐dimensional face is rotated into this new coordinate system. The output provides the corrected 3D face information. This correction step can be iterated several times to achieve a more precise pose correction.
3. Next, these 3D vertices represent points in three‐dimensional space with X, Y, and Z coordinates, rather than a conventional 2D image format used in CNN networks. Specifically, we project these 3D vertices onto the X–Y plane with a defined resolution of 0.1 cm. The *Z*‐axis value for each vertex is retained through the use of *z*‐buffering and scan line algorithms. As a result, the resulting 2D representations of the 3D information are referred to as “depth images.” The outputs of this process are a 2D texture image and a depth image. In the depth image, each pixel *D*_*i,j*_ value represents the *Z*‐axis height of pixel *T*_*i,j*_ in the 2D texture image.
4. Finally, all background areas of the images are cropped to retain only the face, after which they are resized to dimensions of 224 × 224 pixels. The output of each sample comprises a 224 × 224 texture image (three channels corresponding to RGB) and a 224 × 224 depth image. These 224 × 224 images are then utilized for model training purposes.

### 4.3 Model training

The stroke and control samples are randomly split into 10 sections, followed by copying stroke samples three times and flipping each image from left to right as data augmentation. For 10‐fold cross‐validation, the data was divided into 9 training and 1 test sets, and parameters were optimized for the AUC of the aggregation of all 10 folds of the test sets. Xception, Resnet50, VGG19, and EfficientNetB1 models are trained based on texture images with an epoch of 20, respectively. We first drop the last full connection layers and initialize the other layers with pretrained ImageNet weights as transfer learning. Then we add the same structure of the last layers of each network but only set the classification nodes as 2. All deep learning network operations are deployed by the Keras module in Tensorflow 2.4. Here, we utilize the Adam optimizer with a learning rate of 0.0001 to optimize the loss function of binary cross‐entropy. As each sample has gone through data augmentation, probability of images belonging to one sample is averaged to return a final stroke probability under each model. Probability of 0.40 was taken as the threshold to classify samples, followed by computing accuracy, precision, false positive rate, and true positive rate. Then ROC is generated and AUC is computed to evaluate model learning ability.

### 4.4 Model ensemble

Considering the model preference of each deep CNN, we used the weighted average of the four models to derive a final probability of stroke. The AUC of each network is summed and normalized to 1 to return the 4 weights as in the formulae below. Then, each output is multiplied by the corresponding weight and summed together:

$$K=1/\sum_{i}^{n=4}\mathrm{AUC}\;i$$

$$\text{Final Probability}=\sum_{i}^{n=4}K\times \mathrm{AUC}\;i\times \text{Probability}\;i$$

### 4.5 Confidence interval

Each sample prediction is a Bernoulli trial, thus obeying a binomial distribution. Gauss distribution is utilized to mimic binomial distribution under large sample size. The confidence interval can be computed as the formula below, in which *N* is the sample size, *Accu* is the cohort accuracy, *z* is the set to 1.64 when significance level is 95%:

$$\mathrm{Int}=z\times \sqrt{\frac{\left(\text{Accu}\times \left(1-\text{Accu}\right)\right)}{N}}$$

### 4.6 Differential pixel map

The RGB values of each pixel are split into three red, green, and blue channels, together with depth channel for the hypothesis test, respectively. Mann–Whitney *U* test followed by Bonferroni correction (*q* < 0.05) is used to identify significantly differential pixels between control and stroke groups.

### 4.7 Grad‐Cam

Grad‐Cam algorithm (Selvaraju et al., 2020) is used to interpret the facial images importance. Each of the trained models is applied to each sample, and then the last convolution kernel from each model is saved and fed to Grad‐Cam to calculate feature importance on that sample. Only importance maps of samples with true predictions are kept. All the feature importance maps of four CNN models are averaged to generate a final heatmap for visualization.

### 4.8 Facial landmarks and features detection

Seventy‐two salient 2D landmarks coordinates of texture images are detected by Python package “face_recognition,” followed by finding corresponding 3D landmarks based on mapping files obtained from the preprocessing step. The pairwise Euclidean distances are computed between each landmark pair. Then, the Spearman correlation coefficient (RCC) is calculated between each feature and stroke probability. The −logP value matrix is clustered by hierarchical clustering and used to order and visualize RCC matrix.

### 4.9 Face dense registration

3D obj information after posture correction in preprocessing step is first aligned with the nose tip and is moved to (0,0,0). 3D obj information is then densely registered to a face template by Meshmonk (White et al., 2019) in Matlab to enable analysis using traditional machine learning. After registration, each sample shares the same number (7906) of vertexes, and each vertex represents the same face location.

### 4.10 Stroke probability projection on 3D facial image

Partial least square regression (PLSR) is applied to model the relationship between CNN model stroke probability and the registered 3D coordinates of the facial images. The number of components is set to 2 in PLSR model. PLSR coefficients on the x, y, or z axis are visualized on the face template.

### 4.11 Clinical parameters analysis

Electronic medical records are retrieved from Ruijin Hospital, and only variables available for more than 40 subjects are used for downstream analysis. Based on CNN model stroke probability, the samples with a probability more than or less than 0.40 are taken as positive or negative groups, respectively. Mann–Whitney *U* test or RCC is utilized to detect significantly differential (*p* < 0.05) clinical markers between the two groups.

### 4.12 Causal inference test

Causal inference test (Millstein et al., 2009) tool R package “cit” (Millstein et al., 2016) is used to infer potential causal interactions between clinical parameters and stroke probability (FDR <0.20). Pearson correlation coefficient (PCC) is further computed between each pair, followed by only keeping *p* < 0.05 for visualization. We separately construct two 3‐layer networks. The first network consists of significant markers → the remaining markers → stroke probability, and the second network consists of the remaining markers → significant markers → stroke probability. Then, the two networks are combined and visualized together.

### 4.13 Perceived and chronological age‐trained CNN aging clocks

As described in our previous study (Xia et al., 2020), based on Jidong Vectra images, we have trained a perceived or a chronological aging clock based on CNNs using either perceived or chronological age. The aging clock computes a prediction age for each sample. The residue between prediction and chronological age is corrected by a locally weighted regression (LOESS) model to rule out age effects and taken as the aging rate estimated by each clock. More details of model training and correction details can be found in previous study (Xia et al., 2020).

### 4.14 Saturation analysis

To test the sufficiency of training sample size for model performance, we randomly select samples from the training dataset and re‐train models, followed by testing the whole independent dataset. We randomly selected stroke samples with a number from 35 to 185 adding approximately 35 samples each time and matched controls for model training.

## AUTHOR CONTRIBUTIONS

JDJH and JY conceived and designed the project. YY, SS, XC, HY, YL, and WY collected the samples. JDJH designed and YW implemented the analysis with help from KM and HZ. YW and JDJH analyzed the data and wrote the manuscript with help from others.

## CONFLICT OF INTEREST STATEMENT

Patent pending (Application No. 202311298431.6).

## Supporting information


Figures S1‐S4.

Table S1.

## Data Availability

Due to the regulations in China and patients privacy, datasets including sensitive facial images and baseline blood tests can not be publicly shared.
