# Supplementary material for: Prediagnosis recognition of acute ischemic stroke by artificial intelligence from facial images
Source: Aging Cell. 2024 Jun 6;23(8):e14196. doi: 10.1111/acel.14196 (PMC11320352; doi:10.1111/acel.14196)
Supplement: Supplementary file 1 — Figures S1‐S4. [file ACEL-23-e14196-s002.docx]

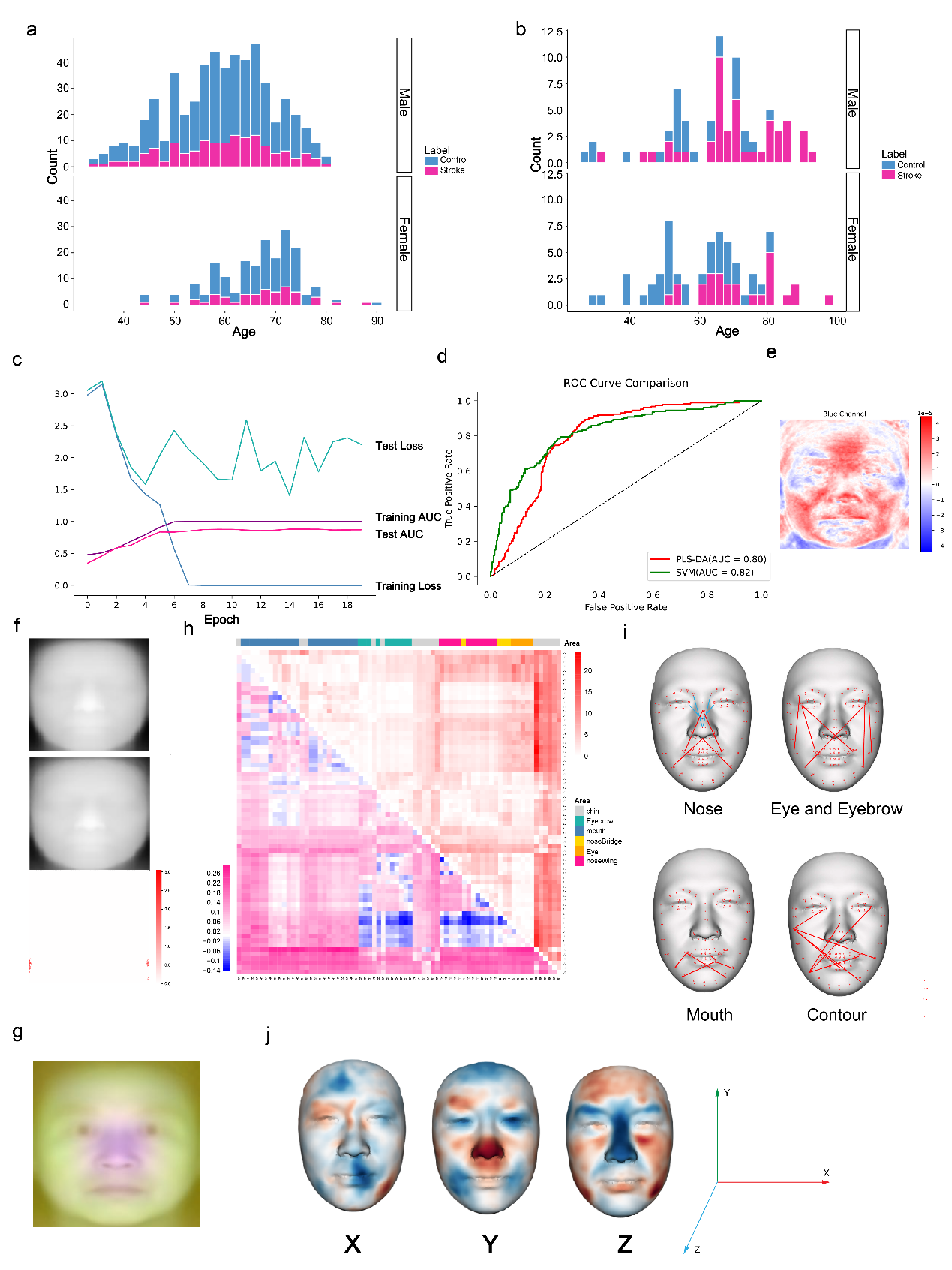


**Supplementary Figure 1.** **The baseline information and model explanation.** **(a and b).** The age and sex distributions of **a.** cross validation cohort and **b.** independent cohort.

**c.** Loss and AUC curves in training and test datasets.

**d.** The ROC curves an AUC values of PLS-DA and SVM model on training datasets.

**e.** The model coefficients visualization of PLS-DA on training datasets.

**f.** Differential pixel map of depth images between control and stroke groups. Color areas indicate the significantly differential pixels. Red color indicates a higher average value in stroke group.

**g.** Average Grad-Cam heatmaps of stroke samples given by the 4 CNNs. Purple color indicates a higher importance while the yellow color indicates a lower importance.

**h.** RCC and FDR (Q values) heatmap of pairwise facial features of 72 landmarks. The upper and lower triangles represent the clustered -logQ values matrix and RCC matrix. The color legend represents the 6 anatomical areas: face contour, nose bridge and wing, mouth and eye brow and eyes.

**i.** Visualization of typical facial features originating from landmarks in 4 anatomical areas.

**j.** X, Y or Z axis coefficients visualization of PLSR model between CNN model stroke probability and X, Y or Z axis values. Red or blue color indicate the coefficients are positive or negative.


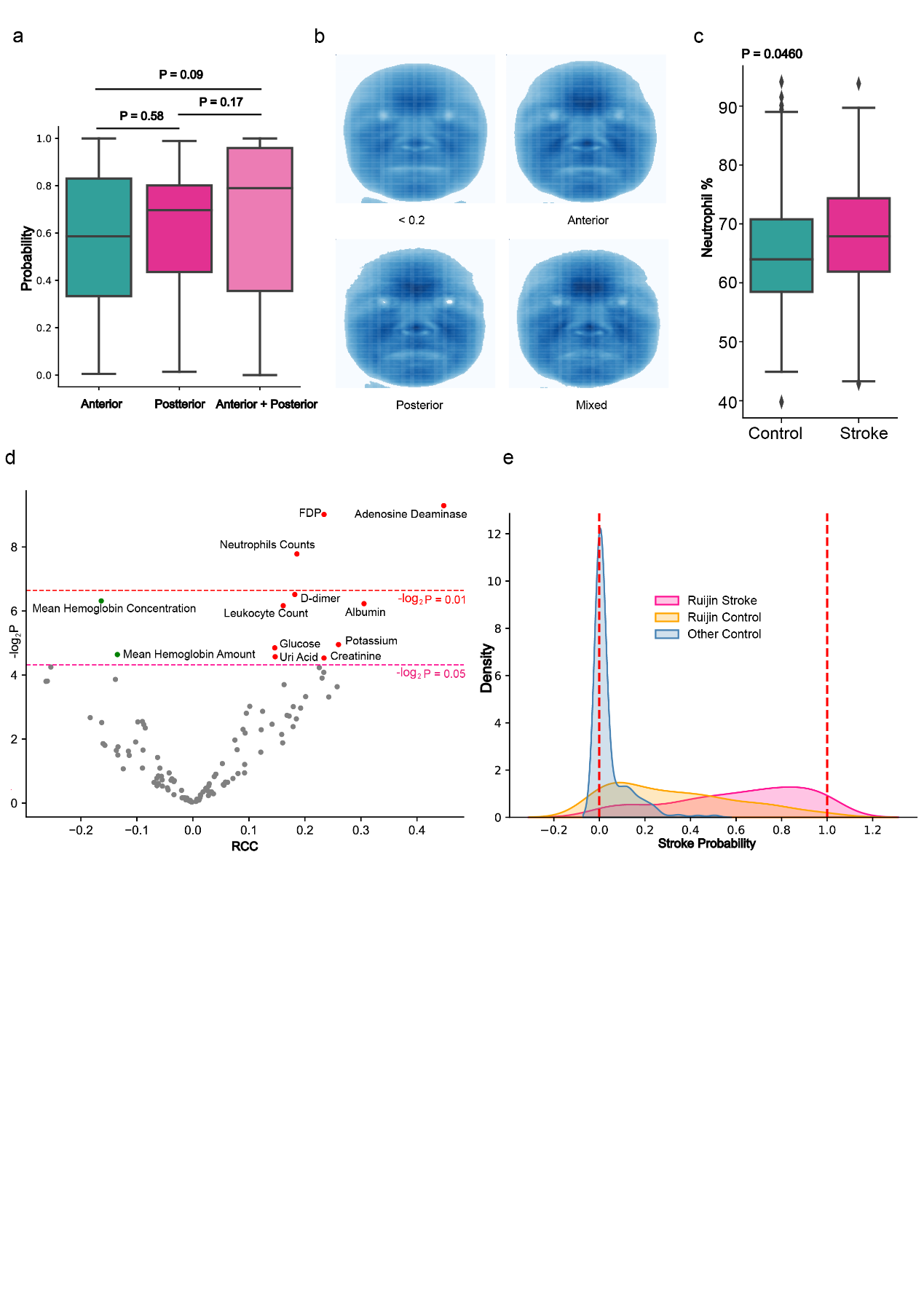


**Supplementary Figure 2.** **Associations of CNN model stroke probability with clinical parameters.**

**a.** The stroke probability distributions of 3 ischemic stroke subtypes show no significant difference as indicated by t-test P values.

**b.** Average blue channel pixel intensity of different stroke subtypes and samples with stroke probability < 0.2 as controls. Pixels with values < 60 are filtered and visualized as white.

**c.** Boxplot of significantly differential marker neutrophil percentage. Control and stroke groups are splited by probability cutoff 0.40. P value is derived by Mann-Whitney U test.

**d.** Spearman correlation coefficients and -logP values between each blood parameter and stroke probability. Red and pink lines indicate the significance threshold 0.01 and 0.05 separately.

**e.** Fitted stroke probability distribution density plot. Pink area indicates the Ruijin stroke samples, yellow area indicates the Ruijin control samples while blue area indicates the other control samples. Vertical dashed line represents the probability threshold to separate positive and negative group.


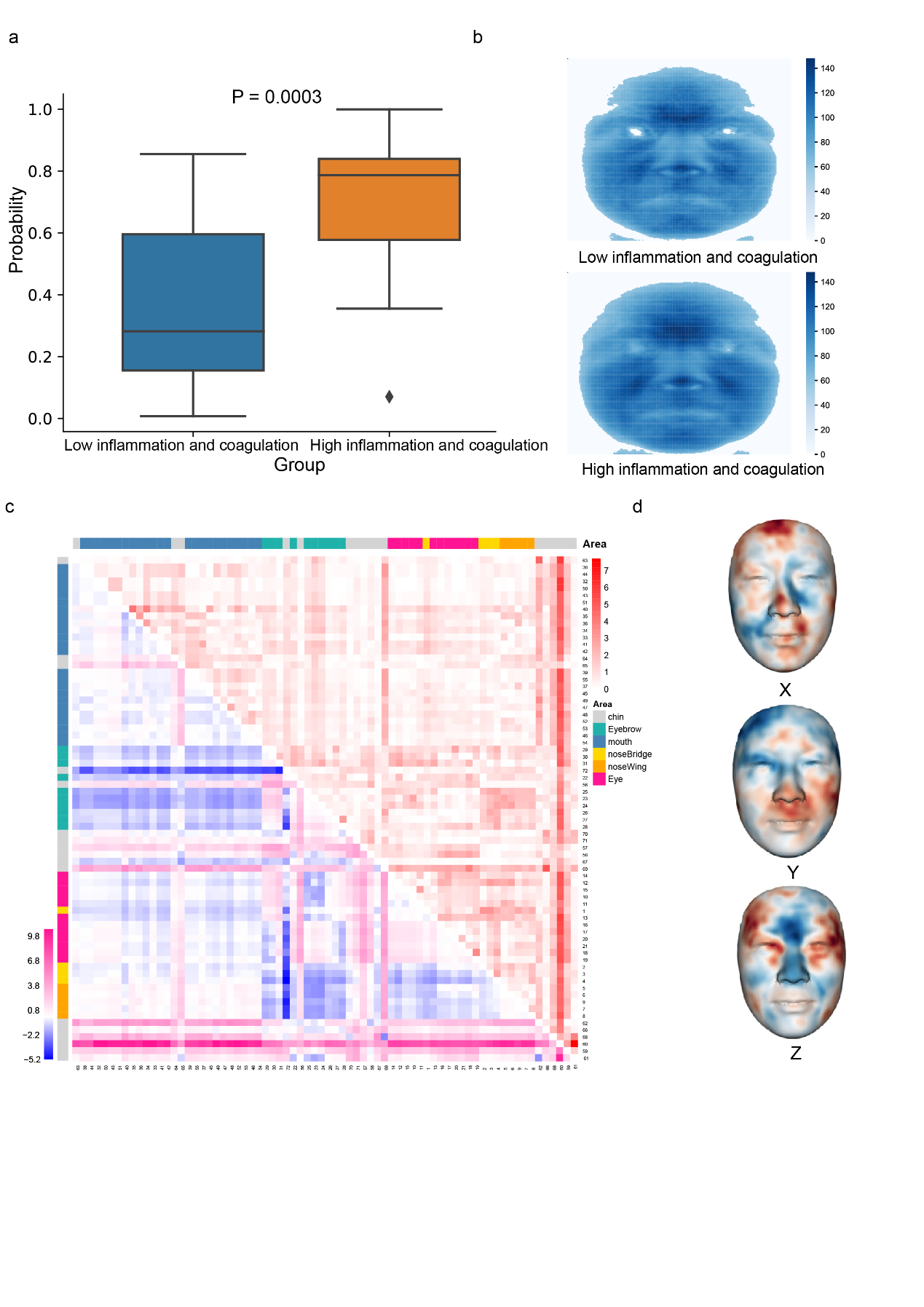


**Supplementary Figure 3. Stroke probability distribution, average blue channel signals and facial features in patients with high and low rank sum of inflammation and coagulation parameters.**

**a.** Stroke probabilities in patients with high (top 25%) and low (bottom 25%) rank sum of inflammation and coagulation parameters (neutrophil counts, glucose and FDP).

**b.** Average blue channel signal of low and high inflammation and coagulation groups.

**c.** The differences (lower triangle) and -logP values (upper triangle) of pairwise distances between 72 facial landmarks between inflammation and coagulation low and high groups. The color legend represents the 6 anatomical areas: face contour, nose bridge and wing, mouth and eye brow and eyes.

**d.** X, Y or Z axis coefficients visualization of PLSDA model with 2 components between the 2 groups and X, Y or Z axis values. Red or blue color indicates the coefficients are positive or negative.


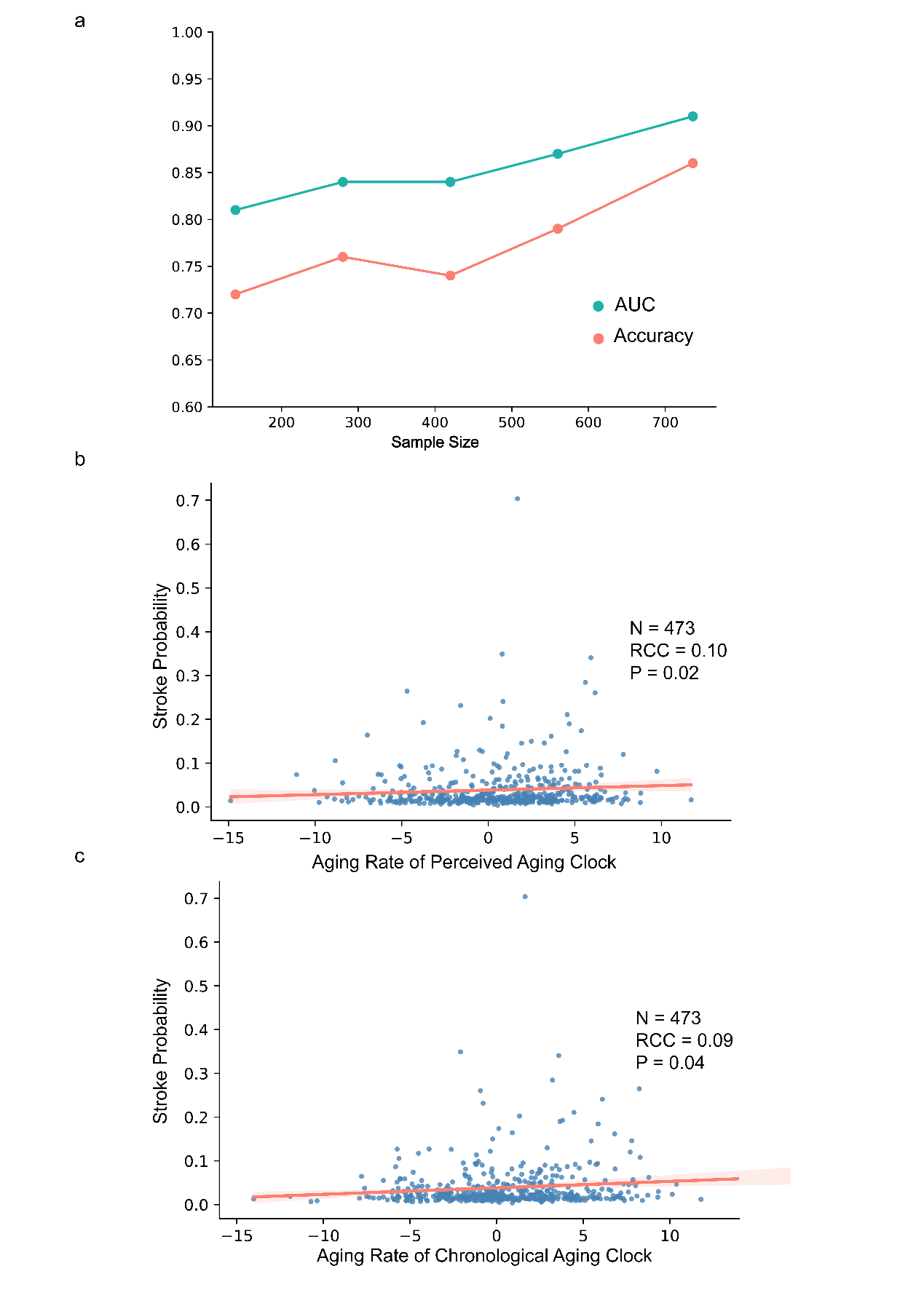


**Supplementary Figure 4. Model saturation analysis and association between stroke probability and aging rate.**

**a.** Sample size saturation analysis of training dataset. Green dots and line indicate test dataset AUC while orange dots and line indicate test dataset accuracy, predicted by models with under a corresponding sample size on the X axis. Stroke samples are random selected from training dataset with matched controls.

**b** and **c.** Association between model computed stroke probability and aging rate derived from CNN aging clock trained by perceived age (b) or chronological age (c) in elderly population (> 50 years) of Jidong cohort.
