# Supplementary material for: Prediagnosis recognition of acute ischemic stroke by artificial intelligence from facial images
Source: Aging Cell. 2024 Jun 6;23(8):e14196. doi: 10.1111/acel.14196 (PMC11320352; doi:10.1111/acel.14196)
Supplement: Supplementary file 2 — Table S1. [file ACEL-23-e14196-s001.docx]

|  | N | Age (AVG±SD) |
| --- | --- | --- |
| Total | 223 | 64.73±12.33 |
| Male | 171 | 62.95±7.94 |
| Female | 52 | 69.04±12.57 |
| MRI positive | 123 |  |
| CT positive | 42 |  |
| Confirmed by CT or MRI | N |  |
| Anterior circulation Stroke^1^ | 78 |  |
| Posterior Circulation Stroke^2^ | 34 |  |
| 1+2 | 32 |  |
| Symptoms | N |  |
| Acute ischemic stroke | 223 |  |
| Acute onset | 223 |  |
| Subjective arm or leg weakness | 143 |  |
| Leg paresthesia disturbance | 134 |  |
| Self-reported speech | 74 |  |
| Subjective facial weakness | 15 |  |
| Dizziness/ Headache | 50 |  |

Supplementary Table 1. Baseline information of acute ischemic patients.
